# Association between Gastrointestinal Diseases and Migraine

**DOI:** 10.3390/ijerph19074018

**Published:** 2022-03-28

**Authors:** Jemin Kim, Sujin Lee, Kiyon Rhew

**Affiliations:** College of Pharmacy, Dongduk Women’s University, 60 Hwarangro-13gil, Seongbuk-gu, Seoul 02748, Korea; 20161780@dongduk.ac.kr (J.K.); 20171734@dongduk.ac.kr (S.L.)

**Keywords:** migraine, GI diseases, gut–brain axis, IBD, IBS, PUD, dyspepsia

## Abstract

Migraine is a common disease worldwide, and recent studies showed that the incidence of migraine was increased in patients with gastrointestinal (GI) diseases. In addition, preclinical evidence suggested a bidirectional relationship between the GI nervous system and the central nervous system called the gut–brain axis. This study aimed to determine the association between several high-prevalence GI diseases and migraine. Patients diagnosed with migraine or GI diseases were classified as the patient group at least twice a year. We included peptic ulcer disease, dyspepsia, inflammatory bowel disease, irritable bowel syndrome, and gastroesophageal disease as GI diseases. A total of 781,115 patients from the HIRA dataset were included in the study. The prevalence of migraine was about 3.5 times higher in patients with one or more GI diseases after adjusting for age, gender, and insurance type (adjusted odds ratio (OR_adj_ = 3.46, 95% CI: 3.30–3.63, *p* < 0.001). In addition, the prevalence of migraine was increased as the number of comorbid GI diseases increased. The prevalence of GI disease was also higher in patients with medication for migraine, both preventive and acute treatment, compared to patients with either acute preventive or acute treatment. There was a statistically significant association between the prevalence of GI diseases and migraine, and the higher the number of accompanying GI diseases, the higher the correlation was in patients using both preventive and acute treatment drugs for migraine.

## 1. Introduction

Migraine is characterized by acute attacks of moderate-to-severe headaches lasting 4 to 72 h accompanied by nausea and/or light sensitivity and sound sensitivity [1]. Migraine is common, affecting more than 100 million people worldwide [2], and the prevalence of migraine in Korea is over 6% [3]. Migraine reduces productivity at work or school and often cause the significant impairment of daily life [4]. In particular, when acute migraine episodes occur frequently, the quality of life is significantly reduced, and preventive treatment is required [5].

A Korean study reported that more than 70% of migraine patients had one or more gastrointestinal (GI) disorders [6]. A study conducted in the United States (US) confirmed a 1.6-fold increase in the prevalence of headaches, including migraine, in patients with irritable bowel syndrome (IBS) [7]. A survey study conducted in Norway reported that the prevalence of headaches, including migraine, increased in patients with GI reflux, constipation, and diarrhea even after adjusting for patient characteristics [8]. In contrast, a study conducted on adults in Italy reported that the prevalence of reflux-like or ulcer-like dyspepsia was not increased in migraine patients, but the prevalence of dysmotility-like dyspepsia was increased by 23% [9].

In patients with both migraine and GI disorders, the absorption of migraine medications may be decreased due to GI disorders, or GI symptoms may become more severe in patients taking migraine medications [10]. In addition, it has recently been reported that the relationship between the two diseases was due to the bidirectional relationship between the GI nervous system and the central nervous system called the gut–brain axis [11]. If GI diseases could cause migraine, it may play an essential role by treating GI diseases or through proper intestinal bacterial growth. Preemptive drug treatment or prophylaxis may be implemented in patients at an increased risk of migraine or those with high frequency or severe symptoms. In other words, investigating the association between migraine and GI diseases has an essential meaning in clinical environments.

Therefore, this study sought to analyze the association between several high-prevalence GI diseases and migraine. We also hypothesized that there might be an interaction between GI diseases and migraine, the drug use of migraine, the number of GI disorders a patient has, and the prevalence of migraine. Hence, we analyzed the prevalence of migraine according to the number of accompanying GI diseases and the association between the severity of migraine and GI diseases.

## 2. Materials and Methods

### 2.1. Study Subjects

Most Koreans are enrolled in the universal health insurance system, and all medical information is reported to the Health Insurance Review & Assessment Service (HIRA). In this study, 3% (~1,400,000) of the patients covered by the HIRA were analyzed using a dataset constructed by random stratification based on 5-year interval ages and gender (HIRA-NPS-2018) [12]. The sample dataset provided by the HIRA was cross-sectional claims data and provided after removing identifiable patient information according to Korea’s personal information protection regulations.

We included patients older than 19 years. We classified patients with diseases diagnosed at least twice a year. Therefore, the cases in which migraine or our target GI diseases were diagnosed only once were excluded. In addition, patients who were diagnosed with a disease that could increase the risk of migraine were excluded from the study. The excluded diseases included neuropsychiatric disorders, headaches other than migraine, some cerebrovascular diseases, and some cardiovascular diseases (Supplementary Table S1) [13,14,15,16]. 

### 2.2. Definition of Diseases 

The classification and diagnosis criteria for each disease were as follows. The GI diseases included peptic ulcer disease (PUD), dyspepsia, inflammatory bowel disease (IBD), irritable bowel syndrome (IBS), and gastroesophageal disease. PUDs included gastric ulcer, duodenal ulcer, peptic ulcer of an unspecified site, and gastric jejunum ulcer. IBD included Crohn’s disease and ulcerative colitis, and gastroesophageal diseases included gastroesophageal reflux disease and other esophageal diseases. Patients diagnosed twice or more in a year were defined as those with the disease. All diseases were defined using the Korean Standard Classification of Disease and Cause of Death-7 (KCD-7). The KCD-7 is based on the International Classification of Diseases (ICD)-10 but has been modified and supplemented to reflect the prevalence of diseases in South Korea (Supplementary Table S1).

### 2.3. Classification of Migraine Medication

Migraine medication was classified into acute treatments and preventive treatment. We categorized four medication user groups: (1) both acute treatment and preventive treatment, (2) acute treatment only, (3) preventive treatment only, and (4) no medication used for migraine as defined. Based on the migraine treatment guidelines, the acute treatments included non-steroidal anti-inflammatory drugs (NSAIDs), acetaminophen, opioids, triptans, ergots, and non-specific migraine drugs [17,18]. Preventive treatment for migraine included beta-blockers, angiotensin-converting enzyme inhibitors (ACEi), angiotensin II receptor blockers (ARBs), calcium channel blockers (CCBs), anticonvulsants, and antidepressants (Supplementary Table S2) [19,20]. We also considered it would be meaningful to determine whether there was a difference in the prevalence of gastrointestinal diseases according to the migraine drug use group.

### 2.4. Statistical Analysis

The association between GI diseases and migraine prevalence is presented as the crude odds ratio (OR) and OR adjusted (OR_adj_) using the patient characteristics. The OR and 95% confidence interval (CI) were derived through binomial logistic regression analysis. All statistical analyses were performed using SAS 9.4 (SAS Institute Inc., Cary, NC, USA). This study was approved by the Institutional Review Board of Dongduk Women’s University (IRB No. DDWU2109-01).

## 3. Results

### 3.1. Patient Characteristics

**Figure 1:**
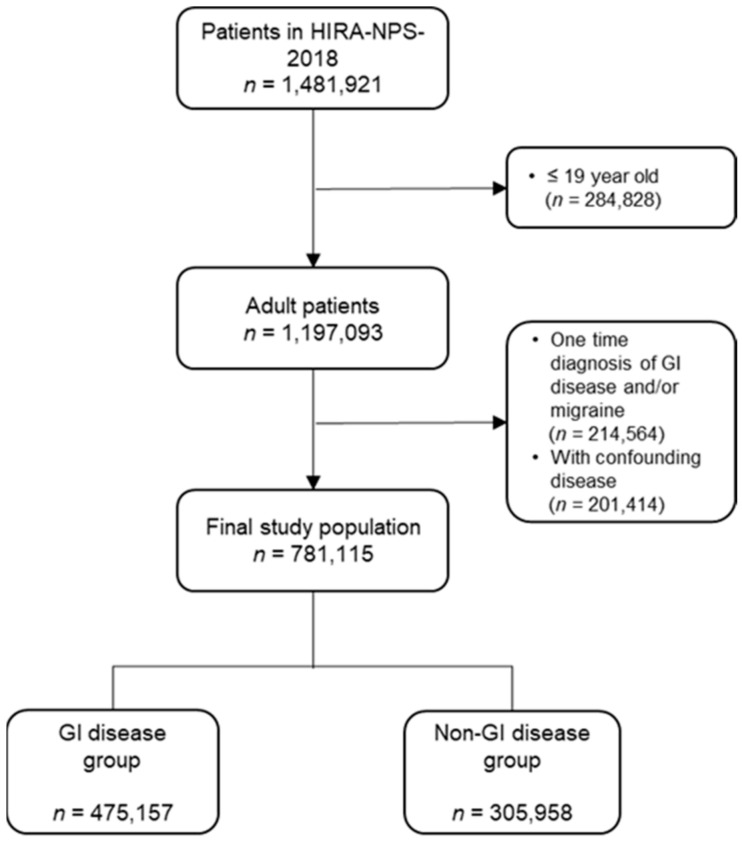
Flow diagram for study subject inclusion. GI: Gastrointestinal.

A total of 1,481,921 patients were included in the HIRA NPS-2018. Patients under the age of 19 years (284,828 patients), with GI diseases and migraine diagnosed only once in a year (214,564 patients), and patients with conditions that increased the risk of migraine (201,414 patients) were excluded. Finally, a total of 781,115 patients were included in this study (Figure 1).

Among the patients included in this study, 305,958 (39.2%) had at least one GI disease. The most common GI disease was gastroesophageal disease (165,140 patients, 21.1%), followed by dyspepsia (150,101 cases, 19.2%), PUD (69,660 patients, 8.9%), IBS (43,184 patients, 5.5%), and IBD (1027 persons, 0.2%). In all gastrointestinal diseases except for IBD, the prevalence was higher in women. A higher prevalence of GI diseases was analyzed in the elderly (≥65 years) than in the adult group (≥20 and <65 years) (Table 1).

### 3.2. Association between GI Diseases and Migraine

In patients with one or more GI diseases, the prevalence of migraine was about 3.7 times higher after adjusting for age, gender, and insurance type (OR_adj_ = 3.46, 95% CI: 3.30–3.63, *p* < 0.001). The prevalence of migraine was significantly increased in all GI diseases except for IBD. The prevalence of migraine was highest in the patients with gastroesophageal diseases (OR_adj_ = 2.576, 95% CI: 2.46–2.69; *p* < 0.001), and the OR_adj_ for dyspepsia, IBS, and PUD were 2.381 (95% CI: 2.28–2.49, *p* < 0.001), 2.176 (95% CI: 2.04–2.33, *p* < 0.001), and 1.998 (95% CI: 1.89–2.12, *p* < 0.001), respectively (Table 2).

According to the number of comorbid GI diseases, the prevalence of migraine increased as the number of GI diseases increased. In patients with one GI disease, the OR_adj_ was 2.924 (95% CI: 2.77–3.08); in patients with two GI diseases, the OR_adj_ was 4.292 (95% CI: 4.03–4.57); and in patients with three GI diseases, the OR_adj_ was 5.710 (95% CI: 5.23–6.24). Patients with all five GI diseases had a 25.69-fold higher prevalence of migraine (95% CI: 3.29–200.57) than those without GI disease (Table 3).

A total of 1919 patients received both acute and preventive treatments, 3757 patients received acute treatment alone, 536 patients received preventive treatment alone, and 2008 patients were not prescribed traditional migraine medications. As a result of the prevalence of GI diseases according to the patient’s drug use for migraine, the highest was in patients who used both preventive and acute treatments (OR_adj_ = 4.669, 95% CI: 4.19–5.20), and the lowest was in the patients without typical migraine medication (OR_adj_ = 2.543, 95% CI: 2.31–2.80) (Table 4).

## 4. Discussion

The prevalence of migraine in patients with GI diseases was 3.46 times higher than in patients without GI disease. In addition, as a result of analyzing individual GI diseases, patients with gastroesophageal diseases, dyspepsia, IBS, and PUD excluding IBD showed a significantly higher prevalence of migraine compared to patients without each GI disease. In the analysis of the number of comorbid GI diseases and the prevalence of migraine, the prevalence of migraine increased incrementally as the number of GI diseases increased. The prevalence of GI diseases was higher in the patients with both acute and preventive treatments than patients with either acute or preventive treatment.

In a study conducted on middle-aged people in the United Kingdom (UK), the prevalence of migraine was significantly increased in patients with IBS or PUD. However, the prevalence was not statistically significant in patients with IBDs (Crohn’s disease or ulcerative colitis) [21]. The prevalence of migraine in IBD patients was increased compared to the controls in some other studies [22,23]. Gastroesophageal disease, dyspepsia, IBS, or PUD, which showed a significant association with migraine, is highly influenced by stress and the external environment. Gastroesophageal diseases, dyspepsia, and IBS have high overlapping rates among GI diseases [24,25]. In a Korean study, non-erosive reflux disease was also found in 74% of the patients with functional dyspepsia [25]. These diseases have common pathophysiological mechanisms, including visceral hypersensitivity, GI motor dysfunction, and nervous system dysfunction [26]. However, Crohn’s disease and ulcerative colitis are chronic inflammatory diseases of the intestinal tract and are mainly induced by the abnormal immune response of serum and mucosal autoantibodies to intestinal epithelial cells [27,28]. This difference in the pathophysiological mechanisms between IBD and other GI diseases may be the cause of different correlations between migraine and individual GI diseases.

GI diseases and migraine have complex but related pathophysiologies, and evidence from a number of GI diseases showed that nerve fibers become more sensitive to pain by nerve signals, endocrine signals, or the immune system, thereby increasing the risk of migraine. The prevalence of migraine increased as the number of accompanying GI diseases increased. The 95% confidence interval in patients with five GI diseases was wide due to the small number of patients in this group, but the trend was maintained. In patients with multiple overlapping GI diseases, abnormal signal transmission through the gut–brain axis was more intensified, and the prevalence of migraine was likely to be higher. Previous studies analyzed and reported a correlation between individual GI diseases and the prevalence of migraine, but the prevalence of migraine according to the number of comorbid GI diseases can also provide meaningful information.

Migraine medications are divided into those for acute episodes and those for prevention. Patients take medication for acute attacks immediately after the onset of a migraine or in the presence of a migraine aura. If the migraine causes significant disturbances in daily life or the headaches appear more than four days a month, patients are recommended to take preventive medication. According to migraine medication use, the prevalence of GI diseases was the highest in the group using both acute and preventive treatments. We found that the group diagnosed with migraine and did not use additional migraine drugs had the lowest prevalence of GI diseases, followed by the group using only prophylactic therapy, the group using only therapeutic agents, and the group using both prophylactic and acute treatments. These results indicate that the prevalence of migraine and GI diseases were correlated.

We confirmed that the prevalence of GI disease in patients with both acute and preventive treatment was higher than other medication use groups. The results might suggest that the severity or frequency of migraine episodes could affect GI diseases’ prevalence. In addition, the association could be further strengthened by interactions between the drugs used to treat migraine and GI diseases. GI motility may decrease during acute migraine attacks, and drug absorption may also be impaired. Medication compliance for migraine may decrease due to nausea or GI disease, and GI symptoms or diseases may worsen due to the adverse drug reactions of the drug itself. For example, NSAIDs used to treat migraine headaches may cause esophagitis or increase the risk of gastroduodenal ulcers, bleeding, and perforation, increasing the risk of NSAID-induced GI disease. In addition, in GI diseases, drug absorption is limited, so the effectiveness of migraine preventive or therapeutic drugs may decrease.

There are several hypotheses regarding the interaction between migraine and GI disorders. First, there is the theory of inflammatory mediators. Immune cells and inflammatory mediators in the gut induce visceral pain, and the serum levels of these tumor necrosis factors and proinflammatory cytokines such as Interleukin 1β increase during seizures in migraine patients [29]. Therefore, inflammatory signals interact through the gut–brain axis and can cause both GI diseases and migraine. The second hypothesis involves a correlation between the two diseases mediated by the role of neuropeptides. Glutamate and neuropeptide Y (NPY), neurotransmitters linking the gut–brain axis, are elevated during migraine attacks and play a role in migraine pathophysiology [30]. These substances are also involved in the pathogenesis of GI disorders. For instance, calcitonin gene-related peptide (CGRP), one of the main biomarkers of migraine, is secreted from the trigeminal nerve during a migraine attack and dilates blood vessels, causing a migraine [31]. CGRP may cause abnormal gastric relaxation control, resulting in functional dyspepsia due to gastric dysfunction [32]. When the blood level of CGRP is high, it may contribute to the development of GI diseases due to the systemic action of CGRP. Another hypothesis is the correlation between the two diseases via the serotonin pathway. Because serotonin concentrations are decreased during migraine attacks, these changes can lead to migraine, dilating blood vessels in the brain or causing inflammatory changes [33]. Therefore, selective serotonin reuptake inhibitors (SSRIs) are used to treat or prevent migraine. Serotonin activates a neurotransmitter in the enteric nervous system and influences intestinal motility and inflammation [34,35]. Thus, the serotonin pathway can be a target for treating patients with both diseases [11].

This study had several limitations. This study was a cross-sectional study, and rather than suggesting a causality between migraine and gastrointestinal diseases, only a correlation was reported. In addition, because the disease was defined based only on the diagnosis in claims data, the prevalence of migraine was not evaluated according to the severity or duration of each GI disease. Finally, peptic ulcers and some GI diseases could occur due to migraine medications, and drug–disease interactions were overlooked. Nevertheless, this study had several strengths. First, this study presented an association between various GI diseases and migraine in a large number of adult patients using national data from the HIRA. Second, a more objective hypothesis was presented by analyzing the prevalence of migraine according to the number of GI diseases and the prevalence of GI diseases according to migraine drug use. These are the first presented research results to the best of our knowledge. They provided additional information suggesting an association between the prevalence of migraine and GI diseases.

## 5. Conclusions

There was a statistically significant association between the prevalence of GI diseases and migraine, and the higher the number of accompanying GI diseases, the higher the correlation was in patients using both preventive and acute treatment for migraine. In the future, it will be possible to clarify the pathophysiology of the two diseases further to provide a more precise mechanism of the correlation. If these results raise awareness of the risk of co-morbidity of the two diseases and are applied to the prevention or treatment of both diseases in clinical practice, it will improve the quality of life of patients.

## Figures and Tables

**Table 1:** Patient characteristics according to gastrointestinal diseases.

Patients’ Characteristics	Frequency (%)		OR (95% CI)	*p* Value
	No GI Diseases (*n* = 475,157)	GI Diseases (*n* = 305,958)		
Age group				
Adult (≥20 and <65)	428,377 (90.15)	245,931 (80.38)	1 [Reference]	
Elderly (≥65)	46,780 (9.85)	60,027 (19.62)	2.24 (2.21–2.26)	<0.001
Gender				
Man	256,773 (54.04)	132,822 (43.41)	1 [Reference]	
Woman	218,384 (45.96)	173,136 (56.59)	1.53 (1.52–1.55)	<0.001
Insurance type				
Health insurance	467,085 (98.30)	297,603 (97.27)	1 [Reference]	
Medical aid	7960 (1.68)	8242 (2.69)	1.63 (1.58–1.68)	<0.001
Veterans’ welfare	112 (0.02)	113 (0.04)	1.58 (1.22–2.06)	<0.001

GI: Gastrointestinal, OR: odds ratio, CI: confidence interval.

**Table 2:** The association between migraine and gastrointestinal diseases.

GI Diseases		Frequency (%)		OR (95% CI)	*p* Value	OR_adj_ (95% CI)	*p* Value
		No Migraine (*n* = 772,677)	Migraine (*n* = 8438)				
GI diseases	No	472,771 (61.19)	2386 (28.28)	1 [Reference]		1 [Reference]	
	Yes	299,906 (38.81)	6052 (71.72)	4.00 (3.81–4.19)	<0.001	3.46 (3.30–3.63)	<0.001
Subgroups of GI diseases							
PUD	No	704,568 (91.19)	6887 (81.62)	1 [Reference]		1 [Reference]	
	Yes	68,109 (8.81)	1551 (18.38)	2.33 (2.20–2.46)	<0.001	2.00 (1.89–2.12)	<0.001
Dyspepsia	No	625,889 (81.00)	5125 (60.74)	1 [Reference]		1 [Reference]	
	Yes	146,788 (19.00)	3313 (39.26)	2.76 (2.64–2.88)	<0.001	2.38 (2.28–2.49)	<0.001
IBS	No	730,535 (94.55)	7396 (87.65)	1 [Reference]		1 [Reference]	
	Yes	42,142 (5.45)	1042 (12.35)	2.44 (2.29–2.61)	<0.001	2.18 (2.04–2.33)	<0.001
IBD	No	771,666 (99.87)	8422 (99.81)	1 [Reference]		1 [Reference]	
	Yes	1011 (0.13)	16 (0.19)	1.45 (0.88–2.38)	0.137	1.61 (0.98–2.64)	0.061
Gastroesophageal disease	No	611,257 (79.11)	4718 (55.91)	1 [Reference]		1 [Reference]	
	Yes	161,420 (20.89)	3720 (44.09)	2.99 (2.86–3.12)	<0.001	2.58 (2.46–2.69)	<0.001

GI: gastrointestinal, OR: odds ratio, OR_adj_: adjusted odds ratio, CI: confidence interval, PUD: peptic ulcer disease, IBS: Irritable bowel syndrome, IBD: Inflammatory bowel disease. OR_adj_: adjusted odds ratio by patients’ age, gender, and insurance types.

**Table 3:** The association between migraine and the number of comorbid GI diseases.

The Number of Comorbid GI Diseases	Frequency (%)		OR (95% CI)	*p* Value	OR_adj_ (95% CI)	*p* Value
	No Migraine (*n* = 772,677)	Migraine (*n* = 8438)				
0	472,771 (61.19)	2386 (28.28)	1 [Reference]		1 [Reference]	
1	205,809 (26.64)	3411 (40.42)	3.28 (3.23–3.55)	<0.001	2.92 (2.77–3.08)	<0.001
2	71,625 (9.27)	1829 (21.68)	5.06 (5.25–5.85)	<0.001	4.29 (4.03–4.57)	<0.001
3	19,488 (2.52)	676 (8.01)	6.87 (7.22–8.35)	<0.001	5.71 (5.23–6.24)	<0.001
4	2973 (0.38)	135 (1.60)	9.00 (8.55–11.44)	<0.001	7.38 (6.18–8.83)	<0.001
5	11 (<0.01)	1 (0.01)	18.17 (1.84–109.11)	0.005	25.69 (3.29–200.57)	0.002

GI: gastrointestinal, OR: odds ratio, OR_adj_: adjusted odds ratio, CI: confidence interval. OR_adj_: adjusted odds ratio by patients’ age, gender, and insurance types.

**Table 4:** The association between GI diseases and migraine by migraine medication use.

Categories of Medication Use for Migraine	Frequency, *n* (%)		OR (95% CI)	*p* Value	OR_adj_ (95% CI)	*p* Value
	No GI Diseases (*n* = 475,157)	GI Diseases (*n* = 305,958)				
No migraine	472,771 (99.50)	299,906 (98.02)	1 [Reference]		1 [Reference]	
Migraine						
Other medication	648 (0.14)	1360 (0.44)	3.31 (3.01–3.63)	<0.001	2.54 (2.31–2.80)	<0.001
Only preventive treatment	218 (0.05)	536 (0.18)	3.88 (3.31–4.54)	<0.001	3.24 (2.76–3.80)	<0.001
Only acute treatment	1073 (0.23)	2684 (0.88)	3.94 (3.95–4.23)	<0.001	3.56 (3.31–3.83)	<0.001
Prophylactic and acute treatment	447 (0.09)	1472 (0.48)	5.19 (4.67–5.77)	<0.001	4.67 (4.19–5.20)	<0.001

GI: gastrointestinal, OR: odds ratio, OR_adj_: adjusted odds ratio, CI: confidence interval. OR_adj_: adjusted odds ratio by patients’ age, gender, and insurance types.

## Data Availability

The datasets analyzed in the current study are available from the corresponding author upon reasonable request.

## Supplementary Materials

The following supporting information can be downloaded at: https://www.mdpi.com/article/10.3390/ijerph19074018/s1, Table S1: The list of KCD-7 diagnostic codes of diseases in the study; Table S2: Migraine medication classification.

## Abbreviations

ACEi	angiotensin-converting enzyme inhibitors
ARBs	angiotensin II receptor blockers
CCBs	calcium channel blockers
CGRP	calcitonin gene-related peptide
GI	gastrointestinal
HIRA	Health Insurance Review & Assessment Service
IBD	inflammatory bowel disease
IBS	irritable bowel syndrome
ICD-10	International Classification of Diseases-10
KCD-7	Korean standard classification of disease and cause of death-7
NPS	national patient sample
NPY	neuropeptide Y
NSAIDS	nonsteroidal anti-inflammatory drugs
OR	odds ratio
OR_adj_	adjusted odds ratio
PUD	peptic ulcer disease
SSRI	selective serotonin reuptake inhibitor
