# Supplementary material for: Association between Gastrointestinal Diseases and Migraine
Source: Int J Environ Res Public Health. 2022 Mar 28;19(7):4018. doi: 10.3390/ijerph19074018 (PMC8997650; doi:10.3390/ijerph19074018)
Supplement: Supplementary file 1 [file ijerph-19-04018-s001.zip › ijerph-1616277-supplementary.pdf]

Supplementary Information

If the patients have diagnostic code twice or more in a year, we defined the patients had the corresponding disease. Korean standard classification of disease and cause of death-7 (KCD-7) was used for definition of disease. KCD-7 reflects the update of the International Classification of Diseases (ICD-10), and refined the Korean subtype disease and rare disease to improve medical terms. Codes could be in any field of the encounter claim (primary or secondary diagnoses).

Table S1. The list of KCD-7 diagnostic codes of diseases in the study.

Category		KCD-7
Corresponding disease		
Gastroesophageal diseases	Gastroesophageal reflux disease	K21
	Other diseases of esophagus	K22
PUD	Gastric ulcer	K25
	Duodenal ulcer	K26
	Peptic ulcer, site unspecified	K27
	Gastrojejunal ulcer	K28
Dyspepsia	Functional dyspepsia	K30
IBD	Crohn's disease	K50
	Ulcerative colitis	K51
IBS	Irritable bowel syndrome	K58
Migraine	Migraine	G43
Excluded diseases		
Neuropsychiatric disorders	Anxiety disorders	F40, F41
	Bipolar affective disorders	F31
	Depressive disorders	F32, F33
	Sleep disorders	G47
	Epilepsy	G40, G41
Other headaches	Headaches other than migraines	G44, R51
Cerebrovascular diseases	Transient cerebral ischemic attacks and related syndromes	G45
	Vascular syndromes of brain in cerebrovascular diseases	G46
	Cerebrovascular diseases	I60-69
Cardiovascular diseases	Angina pectoris	I20
	Myocardial infarction	I21, I22
	Certain current complications following acute myocardial infarction	I23
	Ischemic heart diseases	I24, I25

PUD: peptic ulcer disease, IBD: Inflammatory bowel disease, IBS: Irritable bowel syndrome.

Table S2. Migraine medication classification.

**Acute treatment**	NSAIDs	Aspirin, Aceclofenac, Celecoxib, Cinnoxicam, Clonixin lysinate, Dexibuprofen, Dexketoprofen, Diclofenac, Epolamine, Etodolac, Etoricoxib, Flufenamic acid, Ibuprofen, Ibuproxam, Indometacin, Ketorolac, Ketorolac tromethamine, Lornoxicam, Loxoprofen, Mefanamic acid, Meloxicam, Morniflumate, Nabumetone, Naproxen, Nimesulide, Pelubiprofen, Piroxicam, Polmacoxib, Pranoprofen, Proglumetacin maleate, Propacetamol, Sulindac, Talniflumate, Tiaprofenic acid, Tolmetin, Zaltoprofen, Tolfenamic acid
	Triptans	Almotriptan, Frovatriptan, Naratriptan, Sumatriptan, Zolmitriptan
	Ergot	Ergotamine
	Others	Acetaminophen, Tramadol
**Preventive treatment**	β blockers	Atenolol, Bisoprolol, Metoprolol, Nadolol, Nebivolol, Propranolol
	ACEi, ARB	Candesartan, Lisinopril, Telmisartan
	CCB	Cinnarizine, Flunarizine, Nicardipine, Nifedipine, Nimodipine, Verapamil
	Anticonvulsants	Carbamazepine, Divalproex, Gabapentin, Levetiracetam, Topiramate, Valproate sodium, Zonisamide
	Antidepressants	Amitriptyline, Clonidine, Doxepin, Fluoxetine, Nortriptyline, Venlafaxine

NSAIDs: Nonsteroidal anti-inflammatory drugs, ACEi: Angiotensin-converting enzyme inhibitors, ARB: Angiotensin-converting enzyme blocker, CCB: Calcium channel blockers.
